# The Influence of Physical Education on Self-Efficacy in Overweight Schoolgirls: A 12-Week Training Program

**DOI:** 10.3389/fpsyg.2021.693244

**Published:** 2021-11-03

**Authors:** Francesca Latino, Stefania Cataldi, Valerio Bonavolontà, Roberto Carvutto, Michele De Candia, Francesco Fischetti

**Affiliations:** Department of Basic Medical Sciences, Neuroscience, and Sense Organs, University of Bari Aldo Moro, Bari, Italy

**Keywords:** BMI, fatness, cognitive function, health, exercise, human movement

## Abstract

The purpose of this randomized controlled study was to investigate the impact of a 12-week physical education (PE) program on the self-efficacy of overweight schoolgirls. We randomly assigned 60 overweight schoolgirls (15–17 years) to either an experimental moderate to vigorous aerobic exercise (∼90 min, three times a week) group (*n* = 30) or a control group (CG) (*n* = 30) that received non-specific regular PE lessons with activities chosen by the curricular teacher mainly focused on team games and sports skills that aimed to achieve general psycho-physical wellness (∼90 min, three times a week). To assess the starting level of students and significant changes reached, at baseline and after training, a battery of standardized assessment motor tests and a psychometric scale (generalized self-efficacy scale, GES) were administered. At the end of the intervention, the experimental group reported a considerable decrease in body mass index (BMI) and a large improvement in self-efficacy (*p* < 0.001). No significant changes were found in the CG. The results suggested that the 12-week moderate to a vigorous aerobic exercise program is an effective weight loss intervention and a vehicle to promote a range of outcomes important to the qualitative growth of adolescents. In fact, it could provide a positive and significant impact on the self-efficacy of overweight schoolgirls.

## Introduction

Obesity, namely a state of excess storage of body fat, is a major public health concern in western countries (Esteban-Cornejo et al., 2020). It is caused mostly by improper lifestyle, i.e., on one side, unhealthy diet, while on the other, low levels of physical activity (Jakicic and Davis, 2011; Piccinno and Colella, 2017). According to WHO data, in the past few years, obesity has doubled in children and tripled in adolescents worldwide. Global estimates indicate that 27.5% of adults and 81% of adolescents do not meet 2010 WHO recommendations for physical activity. Thus, even in the latest guidelines on physical activity and sedentary behavior World Health Organization [WHO] (2020) provides recommendations for children and adolescents on the frequency, intensity, and duration of physical activity, required to offer significant physical and mental health benefits.

The consequences of childhood overweight and obesity are very alarming since it represents a condition that causes serious health damage (Włodarczyk and Nowicka, 2019). It is a major risk factor for the onset of metabolic, functional, psychosocial, or quality of life impairments, since it represents an important factor related to falls in obese adults, as it negatively impacts balance and postural sway (Bianco et al., 2014; McKelvey et al., 2019). Moreover, the most worrying aspect of this is that pediatric obesity may compromise social behavior by influencing both the relationship with the body of an individual and the others (Puhl and Suh, 2015).

Several studies have frequently documented that fatness and a higher body mass index (BMI) appear to be associated with lower self-efficacy (Bagherniya et al., 2018; Rachmah et al., 2019; Schmidt and Pichler, 2020) in adolescents, especially in girls and that physical activity has the potential to stimulate positive behavior changes in this age group (Kumar et al., 2015; D’Elia et al., 2020). Self-efficacy is defined as the belief that one has the ability to successfully engage in a specific behavior, and it is an important predictor of weight control and eating habits (Bandura, 1997; Hopkins and Bennett, 2018).

Levels of self-efficacy influence academic, social, weight control, and significant health habit performance. Individuals are able to engage in extraordinary or irrelevant behavior as a result of fluctuations in their beliefs of personal effectiveness. Those who doubt their ability avoid difficult tasks have difficulty in motivating themselves and work badly to achieve their projects (Salles, 2017). Conversely, a sense of resilient effectiveness promotes socio-cognitive functioning in many different ways. People who believe in their ability consider difficult tasks as challenges and opportunities rather than a threat (Bandura, 2012). People who expressed high levels of self-efficacy in the face of adversity remain focused on the task and think very strategically (Ashford et al., 2010). This efficient way of operating increases performance levels and reduces stress and vulnerability to stress. Their belief in their effectiveness activates and supports the involvement of the cognitive functions required to develop ability (Sheeran et al., 2016). On the contrary, considering themselves inefficient slows down the development of those sub-abilities from which more complex performances depend. Among personal risk factors for the onset of eating disorders in adolescents, the level of self-efficacy seems to take a particular meaning. According to Pont et al. (2017), overweight or obese adolescent girls show low levels of personal resources, such as self-efficacy. Previous findings indicate that adolescent girls engaged in sports activities have a higher self-efficacy than those who do not practice any kind of physical activity, and this is more evident following physical activity that requires greater cognitive involvement (Olander et al., 2013; Dishman et al., 2019). In adolescents, the relationship between female sex, higher weight status, and lower self-efficacy has frequently been documented (Losekam et al., 2010).

It is widely accepted that physical activity has a significant role in physical and psychological health since it is capable to persuade people to embrace an active lifestyle. Several previous studies support the idea that physical activity reinforces the perceived self-efficacy beliefs, which is related to changes in mood at the end of exercise (McAuley et al., 1999; Jerome et al., 2002).

As part of physical activity, a key factor in personal achievement is the belief of how to correctly perform motor skills to attain a certain purpose, namely, perception of own ability of an individual to perform a motor task. Thus, each motor experience fulfilled encourages the perceived sense of self-efficacy that is the confidence regarding the ability to successfully master a skill (Bandura, 1997; Di Battista and Vivaldo, 2015; Colella et al., 2020). Self-efficacy is linked to both the perception of the bodily self and factors that represent motor competence (Babic et al., 2014; Bardid et al., 2016). It concerns the capacity to mobilize cognitive and social resources of an individual to carry out a wide repertoire of motor skills expressible in different contexts and every activity for daily living (Robinson et al., 2015). Latest investigations (Khodaverdi et al., 2015; Dapp and Roebers, 2019) highlight how perceived self-efficacy springs from the quality of both didactic proposals and motor experiences and plays a crucial role in lifestyle changes (Colella et al., 2020).

The literature suggests that self-efficacy increases through physical activity, and that physical activity is an important mediating factor between self-efficacy and the effects of interventions on eating behavior. High self-efficacy is one of the most important indicators and predictors of psychological wellbeing, specifically about the body self-perception dimension (Colella et al., 2009). Despite self-efficacy has been linked to physical activity in overweight and obese adolescent girls, few studies were focused on targeted interventions for increasing self-efficacy and improved body composition related to physical activity in this population (Thomas et al., 2017).

Therefore, to extend the understanding of these connections, the purpose of this study was to investigate the effects of physical education (PE) program on self-efficacy in overweight adolescent girls based on the assumption that exercise could improve it also following a brief intervention. Moreover, this study has the objective of helping educational establishments to realize the significance of PE and its positive influence on physical, emotional, social, and cognitive factors. It would also be desirable that schools considered the possibility of allocating additional resources to promote the engagement of adolescents to regular physical activity because together with the family, school is the main institution to ensure healthy and balanced growth for our children.

## Materials and Methods

### Study Design

We employed a randomized controlled study design to investigate the effects of a 12-week PE program on the self-efficacy of overweight adolescents.

This study was conducted in a high school, and the students took part in extra-curricular structured physical activities. They consisted of 36 lessons of moderate to vigorous aerobic exercises for the intervention group and non-specific regular PE lessons with activities chosen by the curricular teacher mainly focused on team games and sports skills that aimed to achieve general psycho-physical wellness for the control group (CG). Both the interventions were performed for 90 min 3 days per week at the end of the daily school lessons. Training sessions were conducted in the evening from 4:00 p.m. to 5:30 p.m. on Monday, Wednesday, and Friday. The evaluation regarded 36 lessons monitoring the participants at the 1st and 12th week, respectively. Measurements were administered 1 week before training (pretest) and directly after training (posttest). To allow statistically meaningful comparisons between different types of activities, the subjects were classified as participants in activities that shared similar characteristics.

### Participants

A total of 60 schoolgirls, as a convenient sample, was recruited with an age range of 15–17 years (*M* age = 16.13, SD = ± 0.74) from four local high schools.

In this study, participation was voluntary, and all high schoolgirls from the four local schools were eligible to participate. Inclusion criteria were the following: participants had to be a girl with a high BMI, to be able to complete a moderate-to-vigorous intensity aerobic exercise session, current students in one of the four high schools, and able to abstain from all physical activities outside the parameters of the study protocol during test days.

Any students with a normal or low BMI, those with an orthopedic condition limiting their ability to perform exercises, and those unable to abstain from all physical activity outside the confines of the study protocol on the testing days were excluded from this study.

A total of 65 subjects fulfilled the inclusion criteria and were invited to participate. Of those recruited, 60 agreed to be in this study, while five of those declined to participate due to personal reasons. As a result, the final sample consisted of 60 participants, who completed the assessments at baseline and postintervention. We matched participants randomly to one of the two treatment conditions, both included 30 overweight schoolgirls (experimental group, EG; *n* = 30; CG; *n* = 30). *A priori* power analysis indicated that 54 participants were required to detect a medium effect size (ES) (*f* = 0.25 or 0.4) given a coefficient of correlation *p* = 0.80 with 95% power and α = 0.05, using a within-between mixed design. However, to avoid experimental mortality, i.e., the loss of participants that could threaten the validity of the research design, more schoolgirls were recruited. Before the beginning of the intervention, schoolgirls and their parents were summoned in order to illustrate to them the whole plan of treatment, the study characteristics, and a complete explanation about the purpose of the experiment, its contents, and safety issues based on the Declaration of Helsinki. The researchers ensured the anonymity of the participants, and all parents of all participants provided their written informed consent before the study. This study was conducted from September 2019 to December 2019.

### Procedures

The intervention programs were administered at a local public school. Before the first training session, a special briefing was given to provide an explanation of the content of the exercise training program. One week before the beginning of the intervention, participants were led to the school gym to proceed with the anthropometric measurements that included BMI and performed standardized motor assessment tests that were used to determine the starting level of the physical fitness of each participant. A cognitive test was administered to examine the self-efficacy perception of students the following day. After taking the BMI, the researchers selected a sample of 60 students and matched participants randomly to one of the two treatment conditions. The exercise intensity of each training session was monitored using the OMNI scale to respect exertion in the moderate to vigorous physical activity (MVPA) range of a 5 < rate of perceived exertion (RPE) < 8 and to avoid possible differences between training sessions (Utter et al., 2002).

The participants completed each test immediately prior to and following the intervention, at the same time of the day, and under the same experimental conditions to allow pre- and post-testing data connection and to evaluate the effects of the intervention program. Students were tested individually, and each task was explained before the participants started. Testing took 40 min for each participant. The participants wore clothing appropriate to physical activity and sports shoes throughout the intervention program. All measurements for testing and both the intervention programs were instructed, supervised, and performed by two experienced PE teachers, certified by the Italian Ministry of Education. All trials were performed using a standardized test protocol, observing the same conditions.

### Measures

#### Anthropometric Measurements

Anthropometric measurements were obtained following a standard protocol and instruments (Weiner and Lourie, 1981). To decrease the possibility of an error during the measurement, they were performed three times each and recorded after verification by the same expert operator. After a detailed explanation of the protocol, data were collected. The subjects were sent to a private room where the measurements were taken. The height and weight of each student were measured using a digital weight floor scale with a precision of 0.1 kg (Omron HBF-375 Karada Scan, Japan) and a wall meter to the nearest 0.1 cm (Health-o-Meter Professional Wall Mounted Height Rod, PS Medical, China). For measuring weight, the scale was positioned on a flat surface, and the participant removed any coats, heavy sweaters, and shoes. The researcher secured that the participant was in the middle of the platform of the scale with the bodyweight equally distributed on both feet. Height measures were obtained ensuring that the shoes of participants were removed and that they stand with heels together, arms at sides, legs straight, and shoulders relaxed.

The BMI was calculated using the BMI equation, which is the weight of a person in kilograms divided by the square of height in meters. In children, it is converted to BMI percentile to obtain the relative position of the BMI number of the child among children of the same sex and age (Cole et al., 2000). The international (International Obesity Task Force; IOTF) BMI cutoff points were used to assess the child overweight and obesity, which represents the cutoff points by sex in the age group between 2 and 18 years, defined to pass through BMI of 25 and 30 kg/m^2^ at age 18, obtained by averaging data from Asian and Caucasian children (Cole and Lobstein, 2012). BMI data were obtained before and after the samples undergo a physical training program.

#### Motor Tests

The evaluation included four physical fitness tests as shown below:

1. The 20-m multistage fitness test (beep test) is a maximal running aerobic fitness test used to assess aerobic capacity.
2. The push-up test evaluates the strength and resistance of the upper body muscles.
3. Curl-up test evaluates abdominal strength and resistance.
4. Sit and reach, a common measure of flexibility of the lower back and hamstring muscles. It requires three measures, and the score is the average reached by the three distances.

Due to their simple and time-efficient implementation, these tests are simple and quick to perform (Krishnan et al., 2017). Since minimal equipment is required, their use is ideal for the school context. The physical fitness tests were conducted for both the EG and the CG at the beginning and end of the intervention program.

#### Generalized Self-Efficacy Scale

The Generalized Self-efficacy Scale (GES; Schwarzer and Jerusalem, 1995) is a well-validated and reliable questionnaire designed to assess self-beliefs in the competence of an individual to cope with a variety of stressful and challenging demands. The GES was a 10-item questionnaire using a 4-point Likert-type scale (α = 0.86). The perceived sense of self-efficacy assessment required responses ranging from 1 “not at all true” to 4 “exactly true.” It took approximately 10–15 min to fill out (including instruction and practice phase), and it was self-administered. The total scoring system ranges between 10 and 40, with a higher score indicating more self-efficacy.

#### Exercise Training Intervention

The exercise training program for the EG was composed of three diverse typologies of physical activities with different targets. According to their types, physical activities were grouped into flexibility, moderate-to-vigorous aerobic exercise, and games.

The exercise program was standardized with a typical plan beginning with a *flexibility* session (10 min) followed by a *moderate-to-vigorous aerobic exercise* session (40 min), and finally, a *games* session (30 min) (Table 1). Specifically, we exposed participants to a final part of competitive games with the aim to increase motivation and self-efficacy in participants through the pleasure to be active, acceptance of defeat, and full awareness of own ability of an individual. Each exercise session started with a 5-min warm-up and ends with 5-min cool-down phases with an emphasis on continuous movement and minimal standing around to maintain a safe elevated heart rate. Overall, the exercise program was designed to be enjoyable and appealing by allowing participants to use their favorite music during exercise sessions and experiencing a team mentality. Moreover, participants received weekly reports about their levels of physical activity achieved in the previous week.

**TABLE 1:** Weekly offered exercise sessions for the experimental group (EG).

1	Flexibility: 2 sets of 10 repetitions – Sit to stands, kneeling hip-ups, stretch arm forward, hip circle, bridge with alternating outer thigh squeeze, left clam Aerobic exercise: 2 sets of 12 repetitions – Jumping rope, skaters, jumping jacks, alternating lateral lunges, squat punches, step-ups, sit-ups, donkey kicks, straight leg jackknifes, single-leg glute bridges Game: Catch and throw balls

2	Flexibility: 2 sets of 10 repetitions – Standing march in place, standing side kick, arm circles, half roll-down, double-leg kick, twist and reach Aerobic exercise: 2 sets of 12 repetitions – Lateral toe taps, mountain climbers, side reach jacks, alternate V-sits, leg lifts, diagonal squats, scissors, hip drive step-ups, rear-foot-elevated split squats, flutter kicks Game: Freeze/Tag Zone

3	Flexibility: 2 sets of 10 repetitions – Windmills, step jacks, kneeling inchworms, leg swings, single leg extension, bird dog in knee hover Aerobic exercise: 2 sets of 12 repetitions – High knees, criss-cross jacks, x hops, reverse lunges, squats, hip bridges, russian twists, crunches, knee plank, side step-ups Game: Stay focused in rhythm

4	Flexibility: 2 sets of 10 repetitions for all exercise – Lunge and reach, standing saw, alternating step-up, standing roll-down, chest expansion, calf raises Aerobic exercise: Lateral shuffle floor taps, bench runners, sit-outs, punch jacks and knee pumps, standing × crunches, knee push-ups, chair squats, forward lunges, bicycle crunches, wall hip thrusts Game: Up, Down, Stop, Go

5	Flexibility: 2 sets of 10 repetitions – Rolling like a ball, march in place high knee, kneeling slide, open leg balance, roll up, scissor kick Aerobic exercise: 2 sets of 12 repetitions – Banded vertical jacks, fast-feet drops, knee pumps to overhead jacks, straight-leg sit-ups, walking lunges, sumo squats, standing shoulder presses, full plank, leg lifts, bottoms-up lunges Game: Survivor game

6	Flexibility: 2 sets of 10 repetitions – Standing legwork, romanian deadlift, single leg balance, double leg extension, rollback and twist, flyers Aerobic exercise: 2 sets of 12 repetitions – Jumping rope, X mountain climbers, knee to elbow-toe touches, heel taps, reaching oblique crunches, single-leg deadlifts, glute-bridges, push-ups, dumbbell thrusters, marching hip lifts Game: 4 Walls

7	Flexibility: 2 sets of 10 repetitions – Pendulum, mermaid, upper back roll, swimming, control balance, side-to-side rolling Aerobic exercise: 2 sets of 12 repetitions – Running in place, power skips, star jumps, drop squats, woodchoppers, ab rollbacks, triceps overhead extensions, knee back to knee raises, hip thrust single-arm reaches, hamstring rolls Game: Crazy 8’s

8	Flexibility: 2 sets of 10 repetitions – Knees to chest, cross-over, standing quad stretch, seat straddle lotus, seat side straddle, table top Aerobic exercise: 2 sets of 12 repetitions – Fast feet, broad jumps, spiderman, hover leg extensions, bent-leg raises, dumbbell seesaw presses, squat jump, downward-facing dog, supine reverse crunches, cobra Game: Tic-tac-toe

9	Flexibility: 2 sets of 10 repetitions – Lower lift, leg circle, simple side bends, up-down dogs, roller dip, saw Aerobic exercise: 2 sets of 12 repetitions – Jumping jacks, skip, skaters, single-leg sit-to-stands, forward lunge with arm drivers, contralateral limb raises, twisting jump squat, ab roller, step ups, battle rope double arm slams Game: Slap the ball

10	Flexibility: 2 sets of 10 repetitions – Rocker, cork-screw, swan dive, side-to-side rolling, criss-cross Aerobic exercise: 2 sets of 12 repetitions – Single-leg toe touches, butt kicks, plank jacks, alternating curtsy lunges, alternate heel touches, battle rope double waves, bear crawls, bench flutter kicks, bosu ball leg pull-in, clams Game: Builders and bulldozers

11	Flexibility: 2 sets of 10 repetitions for all exercise – Leg sweep-left, leg lift and arm extension, hover leg lift, curl ups, lower back curl, pelvic curl Aerobic exercise: 2 sets of 12 repetitions – Run in place, knee to elbow and jump, toe taps, push-ups and reach, single-arm rows, half burpees, Leg Raises, banded glute bridge, agility ladder drills, bench hops Game: Kingball

12	Flexibility: 2 sets of 10 repetitions – March in place high knee, kneeling slide, open leg balance, roll up, single-leg stretch, single-leg kick Aerobic exercise: 2 sets of 12 repetitions – Fast feet, criss-cross jack, bosu ball mountain climbers, shoulder tap plank, bicep curls, burpees, dumbbell squats clean, agility ladder drills, bench swiss ball, bosu V-ups, crab walks Game: Flag football

The CG started each training session with a brief full-body dynamic warm-up, continued with a sequence of main exercises focused on sports skills and designed to improve general physical wellness, and ended with cool-down exercises. Warm-up included marching in place, wide toe touch, leg swings, arm swings, shoulder rotations, hip rotations, push-ups, lunges, walking jacks, jumping jacks, hip circles, and bodyweight squats. Regarding main exercises, they included the following sports and activities: volleyball, badminton, table tennis, individual, in pairs, and collective exercises, bodyweight exercises, exercises with small training gear, joint mobility exercises, and pilates exercises. Cool-down consisted of a variety of static stretching exercises which included glute stretch, standing quad stretch, side bench stretch, arm-cross shoulder stretch, overhead triceps stretch, lower back stretch, abdominal stretch, and pose of a child. It was important for muscle relaxation and the improvement of joint range of motion.

#### Statistical Analysis

We carried out statistical analyses using SAS JMP Statistics (Version 15.1, SAS Institute Inc., Cary, NC, United States, 2020). We presented data as group mean (*M*) values and SD. Normality of all variables was tested using the Shapiro–Wilk test procedure, and data were checked for assumptions of homogeneity of variances (i.e., Levene’s test). We used an independent sample *t*-test to evaluate group differences at baseline and a two-way ANOVA (group experimental/control) × time (pre/postintervention), with repeated measures on the time dimension, conducted to examine the effect of the training on all examined variables. When “Group × Time” interactions reached significance, group-specific *post hoc* tests, such as paired *t*-test, were conducted to identify the significant comparisons. Partial eta squared (η^2^*_*p*_*) was used to estimate the magnitude of the difference within each group and interpreted using the following criteria: small (η^2^*_*p*_* < 0.06), medium (0.06 ≤ η^2^*_*p*_* < 0.14), and large (η^2^*_*p*_* ≥ 0.14). ES for the pairwise comparisons was determined by Cohen’s *d* and interpreted as small, moderate, and large effects defined as 0.20, 0.50, and 0.80, respectively (Cohen, 1992). Statistical significance was set at *p* ≤ 0.05.

## Results

All participants received the treatment conditions as allocated, and their average adherence (attendance) to intervention sessions was 96.6% (34.8 of 36 actual sessions). No injuries were associated with either training program. The experimental and CGs did not differ significantly at baseline in age, anthropometric characteristics, and psychological measures (*p* > 0.05) (Table 1). Preintervention and postintervention results for all dependent measures are presented in Table 2.

**TABLE 2:** Changes after 12-week aerobic exercise intervention.

	Experimental group (*n* = 30)			Control group (*n* = 30)		
		
	Baseline	Post-test	Δ	Baseline	Post-test	Δ
20 m Multistage fitness	4.33 (0.95)	8 (1.92)†*	3.66 (1.34)	4.63 (0.96)	3.90 (1.06)	−0.73 (0.98)
Push-up	6.26 (1.83)	13.46 (3.44)†*	7.20 (2.51)	5.96 (1.93)	5.70 (1.78)	−0.26 (0.90)
Curl-up	16.33 (3.22)	24.63 (3.28)†*	8.60 (3.19)	15.90 (2.84)	14.46 (3.10)	−1.43 (1.52)
Sit and reach	4.93 (2.30)	9.36 (3.07)†*	4.43 (1.69)	5.10 (2.17)	3.86 (2.64)	−1.23 (1.33)
Generalized self-efficacy scale	21.43 (3.89)	33.10 (4.75)†*	11.66 (4.97)	21.60 (3.69)	20.26 (4.20)	−1.33 (1.53)
BMI	88.19 (4.60)	82.41 (8.57)†*	−5 77 (6.78)	89.23 (1.92)	90.13 (1.84)	0.90 (1.27)

*Values are presented as mean (±SD); Δ, pre- to post-training changes.*

*^†^Significant “Group × Time” interaction: significant effect of the intervention (*p* < 0.001).*

*Significantly different from pretest (*p* < 0.001).

### Motor Tests

A two-factor repeated measures ANOVA found a significant “Time × Group” interaction for the 20-m multistage fitness test (*F*_1,58_ = 126.16, *p* < 0.001, η*^2^_*p*_* = 0.68, large ES), push-up test (*F*_1,58_ = 279.56, *p* < 0.001, η*^2^_*p*_* = 0.82, large ES), curl-up (*F*_1,58_ = 241.55, *p* < 0.001, η*^2^_*p*_* = 0.80, large ES), and sit and reach test (*F*_1,58_ = 207.34, *p* < 0.001, η*^2^_*p*_* = 0.78, large ES). *Post hoc* analysis revealed that the EG made significant increase from pre- to post-test in 20-m multistage fitness test (*t* = 12.14, *p* < 0.001, *d* = 2.21, large ES) and an increase in push-up test (*t* = 17.37, *p* < 0.001, *d* = 3.17, large ES), curl-up (*t* = 14.76, *p* < 0.001, *d* = 2.69, large ES), and sit and reach test (*t* = 14.32, *p* < 0.001, *d* = 2.61, large ES). No significant changes were found for the CG.

### Generalized Self-Efficacy Scale

Statistical analysis revealed significant “Time × Group” interaction for *GES* (*F*_1,58_ = 186.74, *p* < 0.001, η*^2^_*p*_* = 0.76, large ES). The *post hoc* analysis revealed a significant improvement in the score for this variable (*t* = 12.83, *p* < 0.001, *d* = 2.34, large ES) in the intervention group. No significant changes were found for the CG (*p* > 0.05). In this study, Cronbach’s alpha reliability for the GES was α = 0.69.

### Body Max Index

Statistical analysis revealed significant “Time × Group” interaction for BMI (*F*_1,58_ = 92.14, *p* < 0.001, η*^2^_*p*_* = 0.61, large ES). The *post hoc* analysis revealed a significant improvement in physical fitness (*t* = 8.79, *p* < 0.001, *d* = 1.60, large ES) in the intervention group. No significant changes were found for the CG (*p* > 0.05).

## Discussion

The aim of this study was to investigate the relationship between a 12-week PE intervention, BMI, and the perceived sense of self-efficacy among overweight high school girls, based on the assumption that the practice of physical activity may decrease their BMI and increase the perceived sense of self-efficacy, an important factor for reaching personal and social success.

In this study, the results indicate that the 12-week of PE intervention was the most effective in yielding the highest perceived sense of self-efficacy in overweight high school girls, while the non-specific regular PE lessons were the least effective in producing effects that corresponded with the objectives outlined.

The first important finding of this study was the evidence of the efficacy of physical activity programs to increase the perceived sense of self-efficacy. Several studies have demonstrated that a moderate-to-vigorous aerobic exercise can have substantial effects on self-efficacy (Hamilton et al., 2017; Flanagan and Perry, 2018; Garcia-Silva et al., 2018; Brace et al., 2020). McAuley et al. (1991) argued that participating in an aerobic exercise program is likely to bring about increases in psycho-physical conditioning and attendant increases in self-efficacy. Physical exercise influences the psychological status and psychological characteristics, such as self-efficacy, motivation, and self-esteem (Baumeister et al., 2003). In fact, the perceived sense of self-efficacy can be observed as a determinant and a consequence of physical activity (Li et al., 2018).

In the physical activity context, individuals may experience different psychological mechanisms resulting in learning a new skill, engaging in a physical activity task, or successfully self-regulating physical activity behaviors (Brown et al., 1992). In fact, physical activity might influence self-efficacy by self-management strategies, arising from thoughts, goals, plans, and acts (Dishman et al., 2004). This finding suggests that self-management strategies are a possible mechanism by which physical activity influences self-initiated self-efficacy. Bandura (1997) argued that self-efficacy affects behaviors through self-management strategies that involve cognitive, motivational, affective and selection dimensions.

Perhaps, the most striking finding of this study concerned the positive impact that the aerobic exercise program had on the BMI of overweight girls. In fact, results indicated that girls who showed better scores in the GSE pre to post-test were also those who improved their BMI. At the end of the intervention program, the BMI average in the EG was considerably improved, whereas, in the CG, it remained stable or even increased. These findings are consistent with previous studies that demonstrate an association between body weight, self-efficacy, and aerobic physical activity exists (Trost et al., 2001; Ievers-Landis et al., 2019; Morano et al., 2020). The results of these studies support the hypothesis that moderate-to-vigorous aerobic exercise activity is more effective than low-intensity exercise in inducing weight loss (Keane et al., 2017). In particular, the high-intensity exercise would produce the most significant results at inducing weight loss even without a dietary change (Irwin et al., 2003; Slentz et al., 2004; Church et al., 2009; Jakicic et al., 2011). Furthermore, we assumed that it was due to the fact that girls with high levels of a perceived sense of self-efficacy were able to be more flexible, resilient, and efficient in problem-solving and were mainly focused on achieving their objectives and pursued personal success rather than to be concentrated on difficulties encountered (Barrett Holloway et al., 1988; Schwarzer and Warner, 2013; Cassidy, 2015). In fact, girls who developed a sense of self-efficacy were able to develop positive behavior toward weight control. This is because the perceived sense of self-efficacy is able to address the personal actions of girls in high-profile healthy activities and build in a positive way their future. It can be assumed that a higher perceived sense of self-efficacy leads to higher motivation and higher self-esteem that are able to build positive behavior that drives significant changes in eating behavior and consequently in body weight (Carissimi et al., 2017). This result accords with previous literature, whereby girls who practice moderate-to-vigorous aerobic exercise perform better on certain tasks related to personal wellbeing through the improvement of planning, organization, and problem-solving skills (Hillman et al., 2014). Moreover, self-efficacy beliefs influence certain psychological and behavioral factors, such as motivation, choice of activities, level of effort, persistence, and emotional reactions (Pender et al., 2002). In fact, overweight girls who considered themselves self-efficacy participated more promptly, worked harder, persisted longer, and responded better to difficulties than those with less self-efficacy (Cataldo et al., 2013). These results validate the idea that aerobic exercise may influence the healthy eating habits of overweight girls through the mediational role that self-efficacy plays in motivating individuals to reach their goals (Davidson et al., 2010), as previously reported by Kitsantas (2000). The author indicates that higher self-efficacy beliefs in implementing self-regulation strategies are linked to lower body weight, as it happens in the case of eating habits.

Based on what was discussed, it was possible to claim that the results of the previous research support our hypothesis, according to which overweight girls had significantly improved their perceived sense of self-efficacy and consequently their BMI following a moderate-to-vigorous aerobic exercise intervention. Despite the contribution regarding the significant relationship between aerobic exercise, self-efficacy, and decrease in BMI, some limitations were present within this study. First, this study was related to the small sample size (*N* = 60), generating difficulties in recruiting motivated overweight students to participate. Moreover, the sample included only girls recruited from a population of students at local public schools located in a small district. Thus, the results may not be generalizable to students from other institutions or with other demographic backgrounds. Additionally, although the authors tried to select groups normally distributed, that were as similar as possible to fairly compare the experimental with the control, and the equivalence between the two groups for the dependent variables under analysis was not evaluated *a priori*. This might have affected the results.

A second limitation of this study is the lack of psychosocial measures, such as the assessment of enjoyment, motivation, and perceived competence of participants for the activities performed. In addition, the sessions were performed by two different professionals, and thus, it could be assumed that the motivational climate created by the teachers was slightly differentiated.

Another limitation concerned the fact that we did not evaluate the long-term effects of aerobic exercise on BMI and self-efficacy. Future research would need to examine these possibilities to explain these variables. However, despite the limitations, the strengths of this study were represented by the fact that it could provide valuable information regarding the most effective types of activities in eliciting the psycho-physical health of schoolgirls. Thus, these results suggest that it is advisable to offer additional moderate-to-vigorous aerobic exercise lessons in overweight girls bearing in mind that the association between physical activity, self-efficacy, and decrease of fatness provides a unique opportunity to operate simultaneously at different levels, namely, improving both physical and mental health.

## Conclusion

This study suggests that aerobic exercise is an effective weight loss intervention and a vehicle to promote a range of outcomes, important to the qualitative growth of adolescents. In fact, it provided a positive and significant impact on the self-efficacy of overweight schoolgirls and their body fat. In particular, self-efficacy had an immense effect on reaching weight management due to its potential in inducing important changes in the behavior of girls regarding their attention toward health.

The school environment may be the ideal setting to combine physical activity, with health education, and care of body and mind, as well as for combating and preventing childhood overweight and obesity. School is, in fact, together with the family, the main institution to ensure healthy and balanced growth for our children. It is essential that school includes in their programs the appropriate types and levels of physical activity to provide a unique opportunity to intervene in a winning way on the health of students. In this respect, despite academic achievement not being the subject of our study, we observed that girls who showed an improvement in self-efficacy and BMI were those who reported the best school grades.

Based on the data obtained, the authors believed that in order to help overweight girls in developing effective strategies of life management and self-care, much could be performed practically. In fact, it is important to supply the perceived sense of self-efficacy that helps to spark a virtuous cycle that feeds itself. Therefore, it would be advisable for Scholastic Institutions to consider the current scientific evidence providing services to the students, such as a “tutoring service.” It could help students to adopt important changes in their eating and physical activity behaviors and by giving them the means to improve their physical and cognitive health. Therefore, this strategy should guide PE teachers and health educators in designing more effective interventions intended to increase the daily amount of MVPA in overweight school girls.

Thus, further research in relation to self-efficacy and physical activity with regard to sex and body weight is necessary to determine a dose-response effect and the effectiveness of any long-term benefits of aerobic exercise on fatness, self-efficacy, and academic performance.

## Data Availability Statement

The raw data supporting the conclusions of this article will be made available by the authors, without undue reservation.

## Ethics Statement

The studies involving human participants were reviewed and approved by the University of Bari Aldo Moro. Written informed consent to participate in this study was provided by the participants’ legal guardian/next of kin.

## Author Contributions

FL designed the study, conducted the research, carried out the statistical analysis, interpreted the data, and wrote and revised the manuscript. SC collected the data, involved in the bibliographical research, and helped to supervise the intervention program. VB collected the data, involved in the interpretation of data, and revised the manuscript. RC and MD were involved in the data collection and helped to supervise the intervention program. FF coordinated the study, interpreted the data, and revised the manuscript. All authors contributed intellectually to the manuscript, read the manuscript, and approved the submission.

## Conflict of Interest

The authors declare that the research was conducted in the absence of any commercial or financial relationships that could be construed as a potential conflict of interest.

## Publisher’s Note

All claims expressed in this article are solely those of the authors and do not necessarily represent those of their affiliated organizations, or those of the publisher, the editors and the reviewers. Any product that may be evaluated in this article, or claim that may be made by its manufacturer, is not guaranteed or endorsed by the publisher.
